# Bile acids as modulators of gut microbiota composition and function

**DOI:** 10.1080/19490976.2023.2172671

**Published:** 2023-02-05

**Authors:** Anaïs B. Larabi, Hugo L. P. Masson, Andreas J. Bäumler

**Affiliations:** Department of Medical Microbiology and Immunology, School of Medicine, University of California at Davis, Davis, CA, USA

**Keywords:** Bile acids, microbiome, intestinal homeostasis, colonization resistance

## Abstract

Changes in the composition of gut-associated microbial communities are associated with many human illnesses, but the factors driving dysbiosis remain incompletely understood. One factor governing the microbiota composition in the gut is bile. Bile acids shape the microbiota composition through their antimicrobial activity and by activating host signaling pathways that maintain gut homeostasis. Although bile acids are host-derived, their functions are integrally linked to bacterial metabolism, which shapes the composition of the intestinal bile acid pool. Conditions that change the size or composition of the bile acid pool can trigger alterations in the microbiota composition that exacerbate inflammation or favor infection with opportunistic pathogens. Therefore, manipulating the composition or size of the bile acid pool might be a promising strategy to remediate dysbiosis.

## Introduction

The colonic microbiota contains 100-fold more bacteria than any other microbial community in our body ^1^. This large microbial community functions in the catabolism of nutrients that are not broken down and absorbed by host enzymes in the upper gastrointestinal tract. Its size and catabolic activity make the colonic microbiota the principal source of microbial metabolites. Since microbiota-derived metabolites affect many aspects of human health, ^2–7^ changes in the composition of the colonic microbiota are linked to many human diseases.^8^ Understanding the factors that shape the composition and metabolic activity of the colonic microbiota is therefore a primary objective of microbiome research. The main drivers of the composition and function of the colonic microbiota are the diet and the host environment.^9^

One important player the host uses to shape the environment of the colonic microbiota is the epithelial lining. By maintaining the colonic epithelium in a state of physiological hypoxia, the host limits the diffusion of oxygen into the intestinal lumen.^10^ The resulting anaerobiosis drives a dominance of obligately anaerobic bacteria in the colon (reviewed in^11^). Conditions that abrogate epithelial hypoxia increase the diffusion of oxygen into the lumen of the colon, thereby increasing the abundance of facultatively anaerobic bacteria (reviewed in^10^), which is a microbial signature of dysbiosis.^12,13^ A second function of the colonic epithelium is the maintenance of an inner mucus layer that is largely devoid of bacteria, thereby protecting the epithelial surface from the high-density microbial community in the colon.^14^ Erosion of the inner mucus layer exacerbates colitis, which is attributed to increased penetration of bacteria into crypts.^15–17^

However, in addition to the local control of microbial growth orchestrated by the colonic epithelium, the environment of the colonic microbiota is also influenced by host digestive functions of the upper gastrointestinal tract. Among these factors, ileal bile absorption has been shown to have a marked impact on the colonic microbiota composition and function.^18^ Bile is synthesized by the liver and contains as one of its major components the primary bile acids, which are conjugated to either taurine or glycine. After synthesis, bile is stored in the gallbladder before being released in the duodenum during digestion to help in the absorption of dietary lipids and fat-soluble vitamins.^19^ While about 95% of the conjugated primary bile acids are absorbed in the terminal ileum and transported back to the liver to be recycled, approximately 5% pass into the colon. Here, the conjugated primary bile acids are deconjugated by the microbiota to liberate taurine, glycine, and primary bile acids. The latter are further metabolized by the colonic microbiota into secondary bile acids, thereby modifying the composition of the total bile acid pool.^18^ Conversely, bile acids regulate the composition of the microbiota, directly *via* their antimicrobial activity, and indirectly by interacting with nuclear and membrane receptors modulating intestinal homeostasis and immunity.^2,20^

The complex relationship between bile acids and the gut microbiota plays an important role in shaping the microbiota composition and the defense against enteric pathogens. By regulating the bile acid pool, the microbiota promotes the maintenance and renewal of the intestinal barrier ^21^ and controls the maturation of hosts' innate and adaptive immune responses.^2^ Moreover, bile acid-derived microbial metabolites protect against some opportunistic pathogens.^22^ Infection with enteric bacteria can disrupt ileal bile acids absorption and endocrine regulation of bile acids production.^23,24^ Furthermore, infection can favor the expansion of bacterial taxa that use bile acid-derived taurine to produce hydrogen sulfide, a metabolite that inhibits respiration of facultatively anaerobic bacteria.^25^

This review will discuss how the interaction between bile acids and the microbiota shapes the environment in the colon to maintain intestinal barrier function, immune homeostasis, and colonization resistance against enteric pathogens, and how a disturbance in bile metabolism impacts host gut physiology.

## Bile acid metabolism and the microbiome

### The enterohepatic circulation of bile acids

The enterohepatic circulation of bile acids is a finely regulated process of bile acid production in the liver, conjugation to taurine or glycine, storage in the gallbladder, secretion in the duodenum after a meal, reabsorption in the ileum and transport back to the liver to be recycled. This process happens 4 to 12 times per day in humans and ensures the maintenance of bile acid homeostasis.^19^

**Figure 1.**
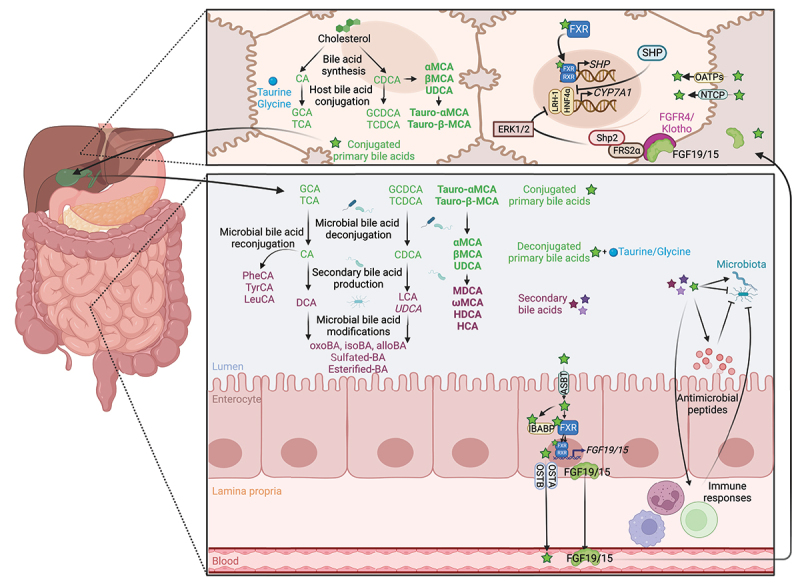
Enterohepatic circulation and bacterial metabolism of bile acids. Primary bile acids, CA and CDCA, are synthesized in the liver from cholesterol.^19^ In rodents, CDCA is converted to α-MCA, β- MCA ^26^ and UDCA,^27^ the latter being considered a secondary bile acid in humans.^28,29^ Mouse-specific bile acids are represented in bold, and human-specific bile acids are represented in italics. Bile acids are conjugated with glycine or taurine (mainly taurine in rodents) before being mixed to the bile, stored in the gallbladder, and secreted to the gut.^19^ In the gut, the microbiota deconjugates primary bile acids to release taurine and glycine.^30^ Conjugated and deconjugated bile acids can be further dehydroxylated,^26^ oxidated/epimerized,^31^ esterified,^32^ sulfated ^33^ and reconjugated ^34^ by the microbiota to produce secondary bile acids. Conversely, bile acids shape the gut microbiota composition, both directly *via* their detergent properties and the ability of the microbiota to metabolize bile acids, and indirectly by modulating hosts antimicrobial peptides production and immune responses.^2,20^ Primary bile acids are absorbed by the enterocytes *via* the ASBT transporter,^32^ transported through the cells by IBABP, and secreted into the blood by the heterodimer OSTα/β.^35^ Bile acids are transported into hepatocytes by NTCP and OATPs, where they activate FXR, which dimerizes with RXR to induce the transcription of SHP to inhibit *CYP7A1* transcription.^36^ In the gut, bile acids activate FXR/RXR heterodimer to induce the transcription of *FGF19/15* that is secreted into the blood to reach the liver. In the liver, FGF15/19 binds to FGFR4/β-Klotho to inhibit *CYP7A1* expression *via* FRS2α, Shp2 and ERK1/2, thus limiting *de novo* bile acid synthesis.^36^ Abbreviations: ASBT, apical bile salt transporter; *CYP7A1*, cholesterol 7α-hydroxylase; ERK1/2, extracellular signal-regulated kinases 1/2; FGF19/15, fibroblast growth factor 19/15; FGFR4/β-Klotho, FGF receptor 4/beta-Klotho; FRS2α, fibroblast growth factor receptor substrate 2α; FXR, farnesoid X receptor; HNF4α, hepatocyte nuclear factor 4α; IBABP, ileal bile acid-binding protein; LRH-1, liver receptor homolog-1; NTCP, sodium taurocholate co-transporting polypeptide; OATPs, organic anion-transporting polypeptides; OSTα/β, organic solute transporter alpha/beta; RXR, retinoid X receptor; SHP, small heterodimer partner; Shp2, Src homology-2 domain-containing protein tyrosine phosphatase-2. Created with BioRender.com.

**Figure 2.**
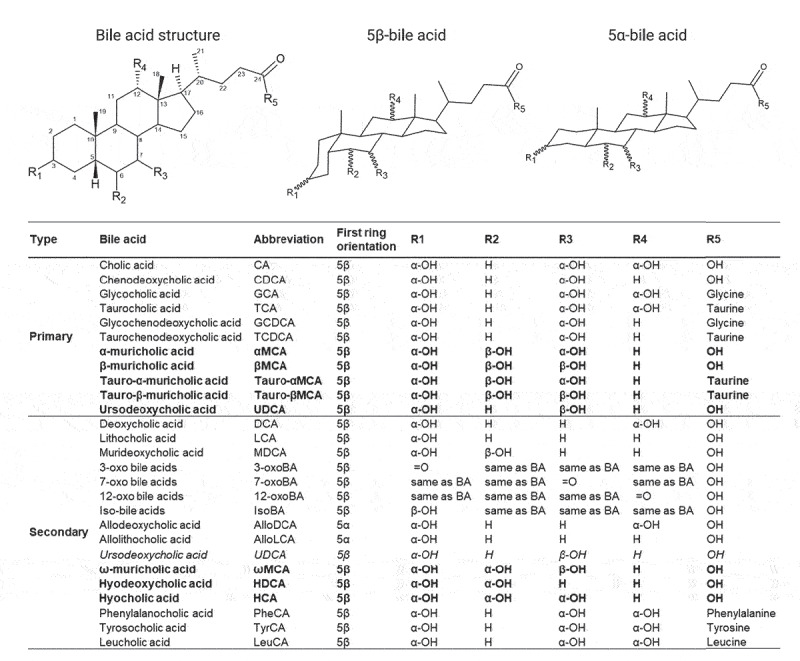
Structure, diversity and metabolism of known human and murine bile acids. Structure and site hydroxylation of the sterol chore for bile acids found in humans and rodents. Hydroxyl groups that are in the α-orientation are located below and are axial to the plane of the sterol chore, while hydroxyl groups in the β-orientation are located above and are equatorial to the plane of the sterol chore. Standard bile acids have the first ring in the β-trans-orientation, yielding 5β-bile acids, while allo-bile acids have this ring in the cis-orientation, yielding 5α-bile acids.^37^ Mouse-specific bile acids are represented in bold, and human-specific bile acids are represented in italics. Created with ACD/ChemSketch, version 2021.1.2, Advanced Chemistry Development, Inc., (ADC/Labs), Toronto, ON, Canada, www.acdlabs.com.

Primary bile acids are synthesized by oxidizing cholesterol, which is catalyzed by cytochrome P450s, with cholesterol 7α-hydroxylase (CYP7A1) being the rate-limiting enzyme (Figure 1).^19^ The predominant primary bile acids synthesized in human hepatocytes are cholic acid (CA) and chenodeoxycholic acid (CDCA).^19^ In rodents, the major primary bile acids are CA, as well as α-, β-muricholic acid (MCA) ^26^ and ursodeoxycholic acid (UDCA)^27^ synthesized from CDCA. Primary bile acids are then conjugated to taurine or glycine to form tauro- and glyco-conjugated bile salts. Conjugated primary bile acids are stored in the gallbladder, before being secreted into the duodenum following food intake to facilitate the absorption of dietary lipids and fat-soluble vitamins through the formation of micelles. In the gut, conjugated primary bile acids are deconjugated and converted into secondary bile acids by the microbiota, thus further increasing the diversity of the bile acid pool (Figure 2).^18^ While small amounts of primary bile acids can be absorbed by passive diffusion, effective absorption requires active transport mediated by the apical bile salt transporter (ASBT) expressed in the ileal epithelium.^36^ Conjugated primary bile acids are mainly transported through enterocytes by the ileal bile acid-binding protein (IBABP).^36^

In enterocytes, primary bile acids bind to the nuclear receptor farnesoid X receptor (FXR), a major regulator of their synthesis, transport, secretion, and absorption.^35^ Primary bile acids differ in their ability to induce nuclear translocation of FXR.^38^ FXR dimerizes with the retinoid X receptor (RXR) to activate the transcription of several genes involved in transcellular transport of bile acids and of fibroblast growth factor (*FGF*) 19 (*Fgf15* in mice) (Figure 1).^35^ The FGF19/15 protein, which is secreted to the portal circulation, travels to the liver where it binds to and activates the FGF receptor 4/β-Klotho (FGFR4/KLB) complex. This complex signals through the docking protein fibroblast growth factor receptor substrate 2 α (FRS2α) and the Src homology-2 domain-containing protein tyrosine phosphatase-2 (Shp2) to stimulate extracellular signal-regulated kinases (ERK) 1/2 phosphorylation, which inhibits activation of *CYP7A1* gene expression mediated by the nuclear factors hepatocyte nuclear factor 4α (HNF4α) and liver receptor homolog-1 (LRH-1), thus reducing bile acids synthesis.^36^ The reabsorbed primary bile acids are mainly exported by the organic solute transporter α/β (OSTα/β) located at the basolateral membrane of the enterocytes into the portal circulation back to the liver.^36^ In hepatocytes, primary bile acids are transported by the sodium taurocholate co-transporting polypeptide (NTCP) and the organic anion-transporting polypeptides (OATPs).^32^ Intrahepatic primary bile acids activate hepatic FXR/RXR heterodimer that regulates the expression of several genes involved in primary bile acid synthesis and small heterodimer partner (SHP) that exerts negative feedback on *de novo* bile acids synthesis by indirectly repressing *CYP7A1* expression.^35^ In total, 95% of the total bile acids are reabsorbed in the ileum and transported back to the liver to be recycled and to regulate their *de novo* synthesis. The remaining 5% reach the colon, where they are metabolized by the gut microbiota and eliminated in the feces.^36^

### Bile acids as integral part of the gut environment

#### Bile acids as part of the microbiome

The term “microbiome” can be defined in an ecological context as a micro-ecosystem including the microbiota and its environment.^39,40^ In the gut, an important component of this environment is bile acids. Conjugated primary bile acids are deconjugated by the microbiota, which yields glycine, taurine, and deconjugated primary bile acids. The latter are further metabolized by the microbiota to form secondary bile acids. As will be discussed below, bile acids and their metabolites shape bacterial growth in the gut. Therefore, to understand what constitutes a healthy microbiome, it is important to consider the status of bile metabolites in addition to analyzing microbiota composition and gene expression.

#### Microbiota changes the bile acid pool

*Directly by metabolizing bile acids*. Microbial transformation of bile acids involves four major pathways: deconjugation, dehydroxylation, oxidation, and epimerization. Moreover, bile acids can be sulfated, esterified, and even reconjugated by the microbiota, thus increasing the diversity of the bile acid pool (Figures 1 and 2).

Deconjugation of tauro- and glyco-conjugated bile salts is the primary step of all subsequent modifications and consists in the cleavage of the glycine or taurine residues to release unconjugated primary bile acids. This reaction mainly occurs in the small intestine and is catalyzed by the microbial bile salt hydrolase (BSH) encoded by the *bsh* gene and found in all major gut microbiota phyla, including *Firmicutes, Bacteroidetes, Actinobacteria*, and *Proteobacteria* and across gut archaea (Figure 1).^30^

Deconjugated primary bile acids can be dehydroxylated at position C-7 of the cholesterol chore by 7α/β-dehydroxylases. The enzymes responsible for this synthesis are encoded by the polycistronic bile acid inducible (*bai*) operon carried by a few bacterial species belonging to the *Clostridium* cluster XIV.^41^ This process converts CA and CDCA into secondary deoxycholic acid (DCA) and lithocholic acid (LCA), respectively, which account for the most abundant secondary bile acids in humans (Figure 1). In rodents, deconjugation and dehydroxylation of the primary bile acids αMCA and βMCA result in the formation of murideoxycholic acid (MDCA).^26^

The bile acid pool can be further diversified by the intestinal microbiota by oxidation and subsequent epimerization at the C-3, C-6, C-7, and C-12 positions of the sterol chore.^31^ These reactions are catalyzed by stereochemically distinct 3-, 6-, 7-, or 12- α-hydroxysteroid dehydrogenases (HSDHs) and β-HSDHs to form oxo- and iso-epimers of these bile acids (Figure 1). They can be performed by an individual bacterial species expressing both α-HSDHs and β-HSDHs or by two species, one expressing an α-HSDH and the other a β-HSDH.^31^ These enzymes have been observed in numerous gut bacteria, including *Bacteroides, Clostridium, Escherichia, Eggerthella, Eubacterium, Peptostreptococcus*, and *Ruminococcus* genera.^31,42^ While UDCA is the primary bile acid in mice,^27^ the production of this bile acid in humans results from the 7α/β oxidation and epimerization of CDCA, performed by C*lostridium absonum*, and therefore is considered a secondary bile acid.^28,29^ Moreover, some human gut bacteria expressing 5α-reductases can transform the classic 5β-bile acid molecules into allo- (or 5α-) bile acid epimers by acting on the C-5 position of the sterol chore.^37,43^ Compared to classical bile acids with a bend structure, these allo-epimers exhibit a planar structure, which is associated with a modification of their physiological effects.^37^ Finally, in mice, 6β-epimerization of the primary bile acid βMCA yields ωMCA,^44^ while subsequent 7β-dehydroxylation or 7β-epimerization of ωMCA yields hyodeoxycholic acid (HDCA) or hyocholic acid (HCA; also known as γMCA), respectively.^45,46^

Other microbiota bile acids metabolism pathways can further increase bile acids pool diversity and regulate bile acids excretion. Sulfation, performed by intestinal bacteria belonging to the genera *Clostridium, Peptococcus, Fusobacterium*, and *Pseudomonas*, is a major metabolic pathway to detoxify and eliminate bile acids by increasing their solubility, decrease their toxicity and intestinal absorption, and enhance their fecal and urinary excretions.^33^ Esterification of bile acids, an activity detected in *Bacteroides, Eubacterium*, and *Lactobacillus*, may increase their hydrophobicity and reduce their solubility (Figure 1).^32^ While the physiological significance of such modifications remains to be investigated, it could minimize their intestinal reabsorption and their toxicity for the microbiota. Recently, a novel set of bile acids has been identified. Instead of taurine and glycine, CA was conjugated with the amino-acids tyrosine, phenylalanine, and leucine (Figure 1).^34^ Studies showed that these compounds are produced by the bacterium *Enterocloster bolteae*, formerly *Clostridium bolteae*, by a mechanism that remains to be elucidated. Although further investigations are needed, these modifications may have a significance in bile acids mediated-intracellular signaling.^34^ Together, these pathways lead to a large diversification of the bile acid pool and regulate bile acids elimination.

Disruption of the microbiota in the case of intestinal diseases impairs bile acid homeostasis. Dysbiosis in the fecal microbiota characterized by an expansion of the *Proteobacteria* (with a strong increase in *Enterobacteriaceae*, including *Escherichia coli*) and a reduced abundance of *Firmicutes* (including *Clostridiales*), is observed in patients with inflammatory bowel disease (IBD) compared to healthy subjects.^47^ This change in the microbiota composition impairs microbial metabolism involved in bile acid biotransformation, leading to an increase in luminal primary bile acids, elevated sulfated bile acids, and a decrease in luminal secondary bile acids.^48–52^ The decrease in secondary bile acids has been associated with reduced relative abundance of members of the microbiota harboring *bsh* genes, in particular *Bifidobacterium* and *Clostridium* clusters IV and XIVb.^53–55^ This is consistent with the dysbiosis observed in IBD patients ^47^ and shows an association between the abundance of *bsh* genes in the microbiome, the composition of the bile acid pool, and the potential repercussion on intestinal health, because a reduced abundance of *Firmicute*-derived *bsh* gene sequences in the microbiota is associated with colorectal cancer.^56^

*Indirectly by regulating hepatic biosynthesis*. By modifying the composition of the bile acid pool, the microbiota influences FXR activation and thus bile acids enterohepatic circulation and hepatic production. Bile acids differ in their ability to activate FXR and follow the order of CDCA > DCA > LCA > CA.^38^ Conjugated versions of these bile acids have an even lower potential to activate FXR, while tauro-αMCA tauro-βMCA, αMCA, βMCA, and UDCA are FXR antagonists.^38^ Gut microbiota depletion in rodents, induced either by antibiotic treatment or germ-free condition, has been associated with an increase in the total bile acid pool compared to conventionally raised animals.^27,57^ Mechanistically, microbiota-depleted mice exhibit an increased proportion of tauro-αMCA, tauro-βMCA, and UDCA. ^27,57^ This leads to a decreased expression and activation of the ileal FXR and its downstream target genes in the ileum, among which *Fgf15*, thus increasing hepatic *Cyp7a1* expression level and *de novo* bile acids synthesis.^27,57^ Gut microbiota depletion also increases bile acid uptake in the colon.^57^ Conversely, the presence of intact microbiota results in a bile acid pool that more potently activates FXR, thereby increasing the ileal expression of *Fgf15* and inhibiting hepatic *Cyp7a1*.^27,57^ In conclusion, whereas an expansion of the bile acid pool results in a feedback repression of *de novo* bile acid synthesis, this regulatory loop is impaired in mice with disrupted microbiota, demonstrating that the microbiota plays an important role in regulating hepatic synthesis of bile acids.

Modulation of gut microbiota composition with chemical compounds or probiotics in mice influences FXR/FGF15/CYP7A1-mediated bile acids synthesis. Administration of tempol, an antioxidant, reduces the abundance of the genus *Lactobacillus* and its *bsh* activity, leading to the accumulation of tauro-βMCA to inhibit FXR signaling.^58^ Similarly, the administration of the VSL#3 commercial probiotic mixture enhances bile acids deconjugation and fecal excretion, and represses the FXR/FGF15 axis, thus increasing hepatic *Cyp7a1* expression and *de novo* bile acids synthesis.^45^ Although these results cannot be directly extrapolated to humans who lack MCA, *in vitro* and *in vivo* studies recently revealed that the abnormal composition of the bile acid pool also limits FXR activation in humans.^59^

#### Bile acids shape the intestinal microbiota composition

The composition and density of bacterial communities is governed by their environment.^9^ Bile acid pool size and composition regulate gut microbial ecology by exerting direct antimicrobial effects on intestinal microbes *via* their detergent properties and indirect effects by inducing the production of antimicrobial peptides and regulating host immunity (Figure 1).^2,20^ The antimicrobial activity of bile acids is demonstrated in rodent models of biliary obstruction and liver injury, which results in small intestinal bacterial overgrowth (SIBO) that can be reversed by administration of bile acids.^60–62^ Rodents fed bile acids and healthy individuals receiving a bile acid analog exhibit significant changes in gut microbiota composition. Administration of taurocholic acid (TCA) or tauro-βMCA to neonate mice shifts the gut microbiota toward a more adult-like composition compared to control mice, demonstrating that bile acids mediate gut microbiota maturation.^63^ In adult rats, the administration of CA-supplemented feed results in a marked expansion of *Firmicutes*, from 54% of the microbiome in control animals to between 93% and 98% in CA-fed animals.^64^ Among *Firmicutes, Clostridium* spp. expanded from 39% to 70% in CA-fed animals.^64^ Conversely, supplementation of mice with DCA decreases *bsh* activity ^65^ as well as the abundance of *Firmicutes*, while increasing the proportion of *Bacteroidetes*. At the genus level, *Parabacteroides* and *Bacteroides* abundance is increased, whereas the abundance of the BSH producers *Lactobacillus, Clostridium XI*, and *Clostridium XIV* is decreased in DCA-fed animals compared to the control group.^66^ In healthy human subjects, the synthetic FXR agonist obeticholic acid (or INT-747) suppresses the synthesis of endogenous bile acids and induces the proliferation of Gram-positive bacteria, including *Streptococcus thermophilus, Lactobacillus casei, Lactobacillus paracasei, Bifidobacterium breve*, and *Lactococcus lactis*, suggesting that FXR activation shapes the intestinal microbiota community composition *via* bile acid-dependent mechanisms.^67^

## Consequences of microbiota-bile acid interactions for gut health

The interaction between the microbiota and bile acids impacts the maintenance of intestinal barrier function, regulates innate and adaptive immunity, and modulates colonization resistance. Effects of bile acids on host cells are mainly mediated by membrane-associated and nuclear bile acid receptors, including FXR, membrane G-protein bile acid-activated receptor (GPBAR)-1, also known as Takeda G protein-coupled receptor 5 (TGR5), nuclear receptor pregnane X receptor (PXR), and vitamin D receptor (VDR). FXR and TGR5 are highly expressed in the liver, the distal ileum, and the colon, in epithelial, endothelial, and immune cells. In addition to the role of FXR in regulating bile acid synthesis, both receptors are essential for maintaining intestinal barrier integrity and limiting inflammation. Bile acids differ in their ability to activate TGR5 and follow the order of LCA > DCA > CDCA > UDCA > CA. PXR is highly expressed in tissues exposed to bile, while VDR is expressed in most tissues, and both preferentially bind LCA.^68^

### Microbiota-bile acids interactions modulate intestinal barrier function

**Figure 3.**
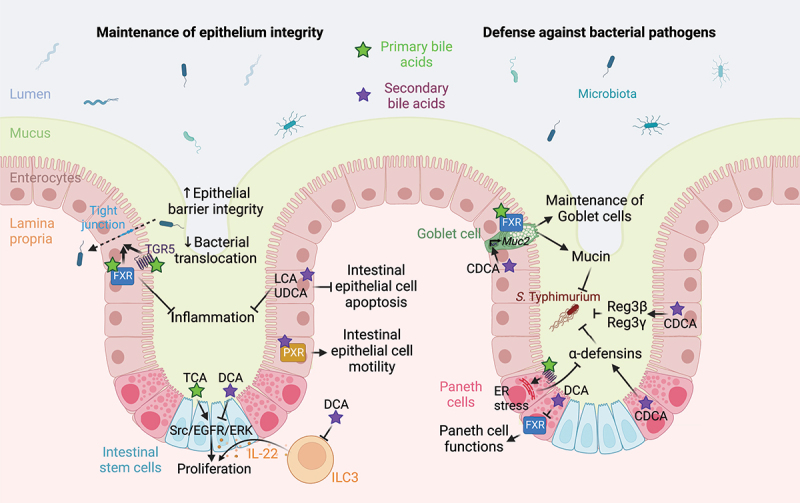
Bile acid-mediated regulation of intestinal barrier function. Bile acids favor the maintenance of epithelial integrity. Indeed, bile acid-mediated activation of TGR5 and FXR receptors in intestinal epithelial cells increases the expression of tight junction proteins, thus improving epithelial barrier integrity and limiting bacterial translocation and inflammation.^60, 72–74^ LCA and UDCA prevents intestinal epithelial cells apoptosis and limit inflammation.^75^ LCA-mediated activation of PXR promotes intestinal epithelial cell motility,^76^ while TCA and DCA modulates Src/EGFR/ERK pathway activation to regulate intestinal stem/epithelial cell proliferation.^77^ DCA also indirectly modulates intestinal stem cells proliferation and differentiation by modulating IL-22-producing type 3 innate lymphoid cells.^78^ Bile acids also promote defense against bacterial pathogens by preventing loss of goblet cells in an FXR-dependent manner ^73^ and favoring *Muc2* expression and mucin production in an FXR-independent manner.^79^ In Paneth cells, primary bile acid-mediated TGR5 activation induces an endoplasmic reticulum stress that limits α-defensins production,^80^ while the secondary bile acid CDCA increases the release of α-defensins by Paneth cells and of Reg3α and Reg3γ by IECs.^79^ However, DCA inhibits FXR in Paneth cells, thus impairing their function.^81^ Abbreviations: CDCA, chenodeoxycholic acid; DCA, deoxycholic acid; ER, endoplasmic reticulum; FXR, farnesoid X receptor; IL-22, interleukin 22; ILC3, type 3 innate lymphoid cells; LCA, lithocholic acid; PXR, pregnane X receptor; Src/EGFR/ERK Src-mediated epidermal growth factor receptor and extracellular signal-regulated kinases activation; TCA, taurocholic acid; TGR5, Takeda G protein-coupled receptor 5; UDCA, ursodeoxycholic acid. Created with BioRender.com.

The ability of intestinal epithelial cells to form tight junctions is critical to the formation and maintenance of the intestinal barrier. Several *in vivo* studies supported the role of bile acids in the regulation of tight junction functions. In mice and rats fed a high fat diet, the increased intestinal permeability and decreased expression of tight junction proteins are associated with an alteration of cecal and plasma bile acid concentration, with an increase in the total bile acid pool and of secondary bile acids.^69,70^ Conversely, administration of the primary bile acid CDCA to prematurely weaned piglets improves intestinal barrier function by inducing zona occludens *ZON-1* expression and limiting *TNFA* (tumor necrosis factor alpha), *IL6* (interleukin 6), and *IL10* gene expression.^71^ Bile acid modulation of intestinal epithelial integrity is mediated by their ability to activate receptors. In mouse and rat models of bile flow obstruction *via* bile duct ligation, the absence of endogenous FXR ligands increases gut permeability and bacterial translocation and reduces expression of the tight junction proteins occludin and claudin 2 (Figure 3).^60,72^ Similar results are observed in mice with a normal bile flow but FXR deficiency.^60^ Conversely, administration of the FXR agonist obeticholic acid to bile duct-ligated rats decreases the severity of intestinal inflammation by increasing tight junction protein expression.^72^ Similarly, in mouse models of chemically induced colitis, activation of FXR limits epithelial barrier permeability and prevents intestinal inflammation.^73^ The role of FXR in intestinal epithelial homeostasis is mediated through FGF proteins. Mice fed a DCA supplemented diet develop dysbiosis, which reduces bile acid deconjugation, thus limiting the activation of the FXR-FGF15 axis and impairing mucosal barrier functions.^66^ When mice on a DCA supplemented diet are treated with the synthetic FXR agonist feraxamine, intestinal injury is reduced, whereas the FXR-FGF15 axis and bile acid homeostasis are restored.^66^ In a mouse model of colitis induced by dextran sulfate sodium (DSS), the administration of an engineered FGF19 protein represses *de novo* bile acid synthesis, modulates the microbiota and the bile acid pool size and composition, favors the maintenance of the intestinal epithelial barrier integrity, and protects from intestinal inflammation.^82^ The anti-inflammatory effects of FGF19 administration are abrogated in DSS-treated mice that are deficient for FXR.^82^ Thus, FXR is required for the protective effects of the FXR-FGF15/19 axis against intestinal inflammation and epithelial disruption, while FGF15/19 complements FXR-mediated maintenance of the intestinal homeostasis by modulating the circulating bile acid pool. In addition, TGR5-deficient mice display an altered expression of tight junctions, an increased intestinal permeability and are more susceptible to chemically induced colitis compared to wild-type mice, suggesting the role of this bile acid receptor in the maintenance of the intestinal barrier.^74^

Bile acids also induce the proliferation of intestinal epithelial cells and limit apoptosis. In mice, the secondary bile acids LCA and UDCA protect from DSS-induced intestinal inflammation and limit epithelial cell apoptosis (Figure 3).^75^ Bile acids promote epithelial regeneration by acting on the TGR5 receptor in intestinal stem cells.^83^ *In vitro*, PXR stimulation by a chemical agonist increases intestinal epithelial cell motility and wound closure.^76^ TCA induces proliferation of intestinal epithelial cells *in vitro via* Src-mediated epidermal growth factor receptor (EGFR) and ERK activation, while DCA inhibits cell proliferation by an FXR-dependent mechanism that may inactivate the Src/EGFR/ERK pathway.^77^ Finally, an increase in DCA induced by a high-fat diet reduces intestinal stem cells proliferation and differentiation by reducing the number of IL-22-producing type 3 innate lymphoid cells, which results in fewer Paneth and goblet cells.^78^

Bile acids also modulate the formation and composition of the mucus layer, which is composed of mucins immersed in antimicrobials, such as defensins. In mouse models of chemically induced colitis, activation of FXR prevents loss of mucin-producing goblet cells.^73^ Mice fed a diet supplemented by CDCA exhibit an increased expression of α-defensins by Paneth cells, elevated transcription of *Muc2* (mucin 2-encoding gene) by goblet cells, and enhanced synthesis of the type-C lectins Reg3β and Reg3γ by the ileal epithelium (Figure 3).^79^ These consequences of CDCA supplementation are linked to an FXR-independent enhancement of resistance against infection with bile-resistant pathogens, including *Salmonella enterica* serovar Typhimurium (*S*. Typhimurium) and *Citrobacter rodentium*.^79^ Conversely, consumption of a high-fat, high-sugar diet increases production of DCA by *Clostridium* spp. in the gut, which in turn inhibits Paneth cell function by acting on FXR and increasing type I IFN signaling.^81^ Similarly, high-fat feeding, by increasing bile acid production and TGR5 expression, induces endoplasmic reticulum stress in the Paneth cells and leads to a decrease in α-defensin 5 and 6, contributing to gut dysbiosis.^80^ The secondary bile acid DCA stimulates, while UCDA inhibits the expression and secretion of human β-defensin-1 and −2 *in vitro*,^84^ which may have implications in the maintenance of intestinal homeostasis.

Finally, VDR may exert a protective function against colon cancer by favoring the detoxification of LCA, a potential enteric carcinogen, in the liver and intestine. However, it is not clear whether this protection is due to vitamin D, LCA, or both.^85^

### Microbiota-bile acids interactions modulate immune homeostasis

Bile acids produced by the microbiota regulate different aspects of immunity, including the induction of inflammatory genes to the recruitment of innate and adaptive immune cells.

**Figure 4.**
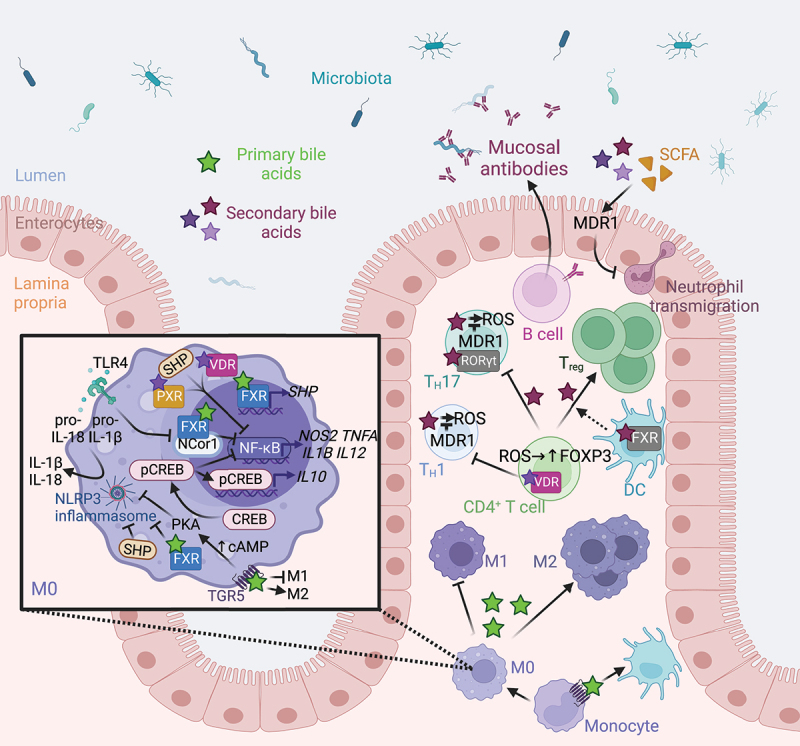
Microbiome-derived bile acids modulates intestinal innate and adaptive immunity. TGR5 stimulation with bile acids promotes the differentiation of monocytes into tolerogenic dendritic cells.^86^ Bile acids promote the polarization of M0 macrophages toward an anti-inflammatory M2 phenotype by stimulating nuclear and membrane receptors.^87^ Indeed, FXR inhibits NF-κB both indirectly by increasing SHP expression ^88^ and directly by binding to the NCor1 complex,^89^ while PXR and VDR receptors directly repress NF-κB-mediated pro-inflammatory gene expression.^90,91^ However, TLR4 activation promotes the release of NCor1 and the activation of NF-κB-mediated gene transcription. FXR, SHP ^92,93^ and the TGR5-cAMP-PKA signaling pathway ^94,95^ repress inflammasome assembly and activation, thus limiting IL-1B and IL-18 production. Finally, TGR5-cAMP-PKA signaling pathway activates CREB phosphorylation, nuclear translocation, and IL-10 production ^87,96^ and inhibits NF-κB-mediated pro-inflammatory gene transcription.^96^ In CD4^+^ T cells, VDR activation inhibits T_H_1 differentiation.^97^ Secondary bile acid epimers promote the differentiation of T_reg_ by blocking FXR in dendritic cells,^98^ and by activating VDR and promoting the formation of ROS in naïve CD4^+^ T cells.^99^ Conversely, bile acid epimers act as an inverse agonist of RORγt in T_H_17 to limit their differentiation. ^42,100,101^ T_H_1 and T_H_17 cell exposure to bile acids drives oxidative stress, which is modulated by MDR1.^102^ Moreover, bile acids and SCFA regulates MDR1 expression to suppress neutrophil transmigration.^103^ Finally, B cells produce mucosal antibodies shaping the gut microbiota.^104^ Abbreviations: cAMP, Cyclic adenosine monophosphate; CREB, cAMP response element-binding protein; FXR, farnesoid X receptor; IL, interleukin; MDR1, Multidrug Resistance Protein 1; NF-κB, nuclear factor-kappa B; NLRP3, Nod-like receptor family pyrin domain containing 3; PKA, protein kinase A; PXR, pregnane X receptor; RORγt, retinoic acid-related orphan receptor gamma t; ROS, reactive oxygen species; SCFA, short-chain fatty acids; SHP, small heterodimer partner; TGR5, Takeda G protein-coupled receptor 5; T_H_, T helper cell; TLR4, Toll-like receptor 4; T_reg_, regulatory T cell; VDR, vitamin D receptor. Created with BioRender.com.

The bile acid receptors FXR, TGR5, and PXR modulate pro-inflammatory gene expression. In chemically induced colitis mouse models, FXR-deficiency worsens, while treatment with the FXR agonist obeticholic acid protects from mucosal inflammation and promotes the expression of antibacterial genes.^73^ Obeticholic acid-mediated activation of FXR decreases TNF-α production in several immune cell populations *ex vivo*.^73^ Mechanistically, FXR regulates the expression of pro-inflammatory genes in macrophages in an SHP-dependent manner by inducing *SHP* transcription. SHP exerts its regulatory effect by inhibiting nuclear factor-kappa B (NF-κB)-mediated activation of the pro-inflammatory gene expression (Figure 4).^88^ Moreover, FXR can regulate the expression of pro-inflammatory genes in an SHP-independent manner by directly binding the promoter of pro-inflammatory genes, including *NOS2* (nitric oxide synthase 2), *TNFA*, and *IL1B*. Binding of FXR to promoter regions stabilizes the nuclear receptor corepressor 1 (NCor1) complex, thus repressing gene expression.^89^ Activation of Toll-like receptor 4 (TLR4) by pathogen-associated molecular patterns leads to the release of NCor1 from the promoters of pro-inflammatory genes, allowing their transcriptional activation.^89^ Finally, PXR and VDR directly inhibit NF-κB signaling, thus reducing pro-inflammatory responses.^90,91^

Bile acids also limit Nod-like receptor family pyrin domain containing 3 (NLRP3) inflammasome activation. FXR and SHP suppress the assembly of NLRP3 inflammasome by physically interacting with NLRP3 and caspase-1 (Figure 4),^92,93^ while activation of the TGR5-Cyclic adenosine monophosphate (cAMP) pathway blocks NLRP3 inflammasome activation by inducing its ubiquitination, which ultimately limits the production of IL-1β and IL-18.^94,95^ Rectal administration of DCA and LCA to various murine colitis models mitigates inflammation, in part by acting on the TGR5 receptor.^105^ Thus, dysbiosis-induced deficiency of secondary bile acids in UC patients may promote inflammation, which could be alleviated by restoring secondary bile acid levels.^105^ Conversely, another study reported that DCA administration in the colon activates the NLRP3 inflammasome in part by stimulating cathepsin B release, which increases IL-1β secretion by macrophages and exacerbates DSS-induced colitis.^106^ Similarly, conflicting results are observed during bacterial infection, where FXR- or TGR5-deficiency decreases inflammasome activation, thereby impairing clearance of *Listeria monocytogenes* or *Escherichia coli*.^107^ Thus, FXR and TGR5 enhance host resistance to bacterial infection by promoting inflammasome-mediated antimicrobial responses in an inflammatory context. However, given the opposite effects reported for secondary bile acids in colitis models, further work is needed to better understand their role during intestinal inflammation.

Bile acids direct the recruitment and differentiation of various immune cells. Compared to wild-type mice, FXR-deficient mice exhibit reduced recruitment of inflammatory cells during DSS-induced colitis.^89^ Mice receiving CDCA supplementation exhibit decreased recruitment of monocytes/macrophages and neutrophils to the intestinal mucosa, whereas the relative amount of B cells is elevated during infection with *S*. Typhimurium and *C. rodentium*.^79^ In macrophages stimulated with lipopolysaccharide (LPS), binding of bile acids agonists to TGR5 activates protein kinase A that phosphorylates cAMP response element-binding protein (CREB). CREB acts as a transcriptional activator of the anti-inflammatory gene *IL10* by directly binding to its promoter,^87,96^ and as a transcriptional inhibitor of pro-inflammatory genes, such as *IL12, IL1B*, or *TNFA*, by reducing NF-κB translocation (Figure 4).^96^ In a mouse colitis model, TGR5 activation shifts the intestinal macrophages from a classical activation status (M1) to an alternative activation state (M2).^87^ In human monocytes, TGR5 activation promotes the differentiation of monocytes into tolerogenic dendritic cells that secrete low levels of TNF-α and IL-12, two cytokines inducing pro-inflammatory T helper (T_H_) 1 responses commonly elevated in IBD patients.^86^ The activation of VDR by LCA reduces T_H_1 cell differentiation *in vitro* by limiting the expression of T_H_1 genes and reducing the production of the T_H_1 cytokines TNF-α and interferon (IFN) γ.^97^ Obeticholic acid-mediated activation of FXR increases the retention of dendritic cells to the spleen, limits splenic regulatory T cells (T_reg_) differentiation, and increases the level of plasma IL-10, thus favoring the amelioration of DSS-induced murine colitis.^108^ Recent studies showed that oxo-, iso-, and allo-epimers of secondary bile acids modulate T cell differentiation. *In vivo*, microbiota-derived isoDCA increases the immunostimulatory properties of dendritic cells by limiting FXR activity, thus indirectly promoting the differentiation of colonic T_reg_.^98^ Furthermore, oxo-, iso-, and allo-epimers of secondary bile acids promote the differentiation of T_reg_ by inducing the production of mitochondrial reactive oxygen species ^100^ and by acting on VDR.^99^ Polymorphisms in the *Vdr* gene significantly increase the risk for developing IBD.^109^ Oxo-, iso-, and allo-epimers of secondary bile acids can also directly bind to and act as inverse agonists of the retinoid-related orphan receptor (ROR) γt, a nuclear receptor mainly expressed by the pro-inflammatory T_H_17 cells,^101^ to limit their differentiation.^42,100^ In mice, oxo-, iso-, and allo-epimers of secondary bile acids alleviate colitis induced by either DSS treatment or by an adoptive transfer of CD4^+^ T cells by modulating the balance between T_H_17 and T_reg_ cells.^99,100^ Numerous studies reported that an imbalance between T_H_17 and T_reg_ compartments contributes to IBD.^110^ The levels of oxo-, iso-, and allo-epimers of secondary bile acids, as well as the levels of microbiome genes required for their synthesis are significantly reduced in the fecal microbiota of IBD patients.^42,43^ Therefore, dysbiosis-induced reduction in the levels of oxo-, iso-, and allo-epimers of secondary bile acids and *Vdr* genetic variants associated with IBD ^109^ might both affect disease susceptibility by altering the T_H_17/T_reg_ balance.

Exposure to bile acids in the lamina propria drives oxidative stress in effector T_H_1 and T_H_17 cells. Effector T cells adapt upon migration to the ileum by upregulating the expression of the xenobiotic transporter Multidrug Resistance Protein 1 (MDR1, alternatively called P-glycoprotein), to limit bile acid-driven oxidative stress (Figure 4).^102^ Bile acids and microbiota-derived short-chain fatty acids (SCFA) act in concert to regulate the expression of *Mdr1* to suppress neutrophil transmigration, thus limiting intestinal inflammation.^103^ A loss of function mutation in *MDR1* has been observed in Crohn’s disease (CD) patients,^102^ while ulcerative colitis (UC) patients display a reduced capacity of the microbiota to induce *MDR1* expression and diminished MDR1 levels, thus supporting the role of the microbiome in regulating mucosal immunity and protecting against intestinal diseases.^103^

Mucosal antibody responses shape microbiota composition, which in turn shape the bile acid pool. B cell deficiency perturbs ileal *bsh* activity of the microbiota, thus altering the bile acid composition in the gut and favoring the development of an ileal enteropathy (Figure 4).^104^

In summary, the ability of the microbiome to produce secondary bile acids constitutes an important factor in the modulation of inflammation and in the recruitment, differentiation, and activation of innate and adaptive immune cells. Conversely, adaptive immunity modulates the microbiota and the production of secondary bile acids. Therefore, maintenance of a balance between these factors is necessary to maintain intestinal homeostasis.

### Microbiota-bile acids interactions modulate colonization resistance

Microbiota confers protection against opportunistic infections through competition for resources and the production of metabolites, such as short-chain fatty acids, that limit bacterial growth.^111^ Microbiota-derived metabolites that limit bacterial growth could be viewed as habitat filters that select for metabolic traits best suited for the environment. Interestingly, the microbiota prevents gut colonization by opportunistic pathogens by cooperating with the host. Specifically, the production of primary bile acids by the host and of secondary bile acids by the microbiota constitutes a habitat filter that strengthens colonization resistance.

#### Microbiota-mediated metabolism of bile acids increases defense against pathogens

The regulation of the bile acid pool by the microbiota plays a role in protecting the host against pathogenic infections. In humans, a higher abundance and activity of *bsh* in the gut microbiota confers an increased resistance against *Vibrio cholerae* infection by degrading the primary bile acid TCA that activates the expression of the pathogen’s virulence genes.^112^ Similarly, *Bifidobacterium bifidum*, by metabolizing GDCA, TDCA, and CA into DCA inhibits the activity of *V. cholerae* type VI protein secretion system (T6SS), a system that injects toxins into competing bacteria, thereby preventing the killing of commensal *E. coli* by the pathogen.^113^ The presence of commensal *Escherichia coli* confers a resistance to *S*. Typhimurium infection by competing for critical resources, such as oxygen,^114^ nitrate ^115^, or iron.^116^

**Figure 5.**
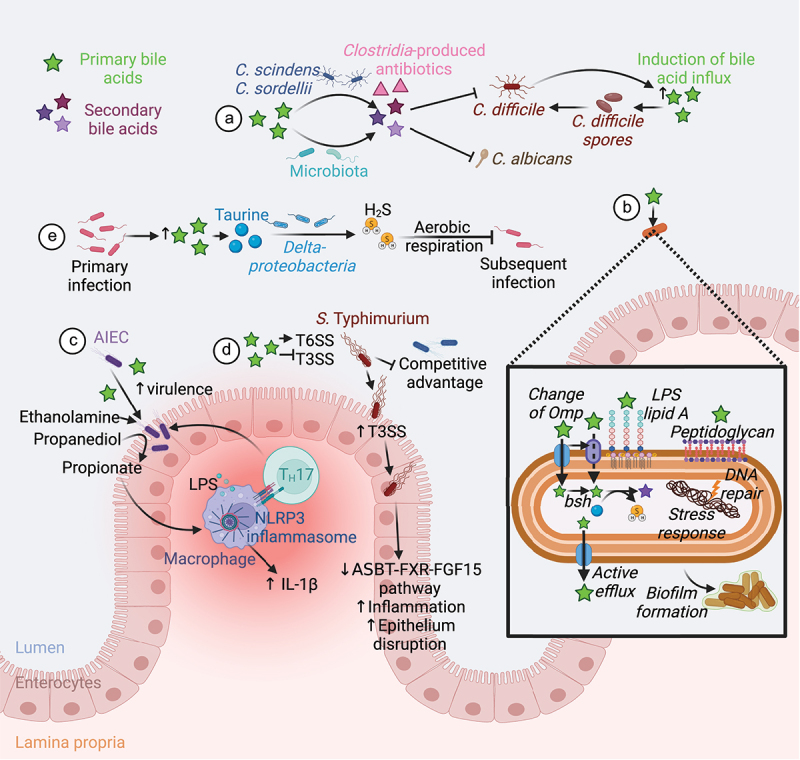
Microbiota-mediated bile acid metabolism and defense against pathogens. (A) Conversion of primary to secondary bile acid by the microbiota confers a colonization resistance against various pathogens. Secondary bile acids exert a direct antifungal activity against *C. albicans*.^117^ The gut members *C. scindens* and *C. sordellii* produce secondary bile acids and antibiotics that act together to inhibit *C. difficile* growth. However, *C. difficile* induces secretion of primary bile acid in the gut to favor its spore germination.^118–120^ (B) Intestinal bacteria and pathogens resist to bile acids by changing the structure of their membrane components (Omp, Lipid A, peptidoglycan), using bile acids as a source of nutrients, actively eliminating bile acids, repairing DNA damage, promoting stress responses, and forming biofilms.^121^ (C) Bile acids promote the expression of AIEC virulence genes ^122–124^ and the use of ethanolamine and propanediol as a nitrogen and a carbon source respectively.^125,126^ Propionate generated from propanediol synergize with LPS to trigger IL-1β production and T_H_17 cell activation, thus promoting intestinal inflammation.^127^ (D) Luminal bile acids increase *S*. Typhimurium T6SS activity and represses its T3SS until the bacteria can reach the epithelium.^128,129^ This confers a competitive advantage to the pathogen in a bile acid-rich lumen.^128,129^ Infection with *S*. Typhimurium decreases the ASBT-FXR-FGF15 pathway and increases inflammation and epithelial disruption.^23,24^ (E) Finally, primary bacterial infection increases the abundance of primary bile acids in the gut. *Deltaproteobacteria* metabolize bile acid-derived taurine to hydrogen sulfide that block pathogen aerobic respiration, thus conferring a resistance to subsequent bacterial infection.^25^ Abbreviations: AIEC, Adherent-invasive *E. coli*; ASBT, apical bile salt transporter; FGF15, fibroblast growth factor 15; FXR, farnesoid X receptor; H_2_S, hydrogen sulfide; IL, interleukin; LPS, lipopolysaccharide; NLRP3, Nod-like receptor family pyrin domain containing 3; Omp, outer membrane porins; T3SS, type III protein secretion system; T6SS, type VI protein secretion system; T_H_, T helper cell. Created with BioRender.com.

Commensal bacteria converting primary bile acids into secondary bile acids provide colonization resistance against *Clostridioides difficile*. Whereas primary bile acids can induce *C. difficile* spore germination, secondary bile acids are toxic to vegetative cells (Figure 5).^118^ Commensal *Clostridia* encoding the *bai* operon protects against *C. difficile* infection by producing secondary bile acids that inhibit *C. difficile* germination, growth, and toxin production.^130^ Moreover, the α-dehydroxylating gut bacteria, *Clostridium scindens* and *Clostridium sordellii*, secrete natural antibiotics that inhibit *C. difficile* growth in the presence of secondary bile acids.^119^ Therefore, disruption of the gut microbiota following antibiotic use constitutes a key risk factor for *C. difficile* infection by altering the bile acid pool.^131^ Antibiotic use also increases the susceptibility to *Candida albicans* colonization in the gut. In susceptible mice, antibiotic treatment favors *C. albicans* growth by altering the gut microbiome and metabolome, including primary and secondary bile acids.^132^ *In vitro*, the secondary bile acids LCA and DCA possess direct antifungal activity against *C. albicans*.^117^ Conversely, the administration of the primary bile acid TCA to antibiotic-treated mice favors the colonization and dissemination of *C. albicans* by altering the microbiota composition and decreasing the number of intestinal mononuclear phagocytes and T_H_17 cells.^133,134^ Thus, modulation of the bile acid pool by the microbiota regulates colonization resistance, either directly by inhibiting pathogen growth or indirectly by modulating mucosal innate and adaptive responses.

#### Microbial strategies to resist bile acids

Gut commensals and enteric pathogens inevitably encounter bile acids. Mechanistically, their amphipathic character and detergent properties directly disrupt bacterial membrane lipid composition, impair macromolecule stability by causing RNA and DNA damages, favor protein misfolding, and limit bacterial access to iron and calcium.^20^ Thus, bile resistance mechanisms, which consist of bile acid metabolism, bile exclusion and extrusion, repair and defense against damages, and modulation of virulence, are crucial to these microbes (Figure 5).^121^

The most important strategy to resist bile acid toxicity is the ability to deconjugate bile acids and reduce bile acid-derived amino-acids. ^135,136^ Some bacteria, such as the pathogens *Brucella abortus* ^137^ and *Listeria monocytogenes* ^138^ express *bsh* as virulence factors. By deconjugating bile acids, BSHs decrease their solubility and emulsifying capacities, thus reducing their toxicity. Probiotic strains of *Lactobacillus* encode multiple distinct BSHs that have different substrate preferences to adapt to specific host niches in the gut depending on bile acid availability.^139,140^ Amino acids arising from deconjugation of bile are metabolized by the gut microbiota. While bacterial metabolism of both glycine and taurine leads to the production of ammonia and carbon dioxide, taurine catabolism results in the additional release of hydrogen sulfide (Figure 5). These products constitute a source of carbon, nitrogen, and sulfur for some bacterial species. *In vitro*, taurine supplementation promotes the growth of *bsh*-expressing *Clostridium* species.^141^ The reduction of CA-derived taurine to hydrogen sulfide favors *Bacteroides* growth by stimulating 7α-dehydroxylation of CA, suggesting that taurine metabolism further increases bile acid degradation.^142^ Consumption of taurine and TCA boosts the expansion of the sulfide producer *Bilophila wadsworthia*^143,144^ and promotes colitis in genetically susceptible IL-10^−/−^ mouse model.^143^

Other mechanisms of resistance are shared between commensals and pathogens. The structure of the bacterial cell envelope constitutes a barrier against bile acid entry, which is used by Gram-negative bacteria to increase their tolerance to bile acids.^145^ For example, certain modifications in the lipid A moiety of lipopolysaccharide are important for the bile resistance of *S*. Typhimurium and *E. coli* (Figure 5).^146,147^ Furthermore, the bile acid tolerance of *S*. Typhimurium involves changes in the peptidoglycan structure ^148^ and expression of very long O antigen chains.^149^ Even though the bacterial envelope limits bile acid uptake, these metabolites can reach the bacterial cytosol through outer membrane porins (Omp), which have different diameters and little substrate specificity. Thus, changes in Omp synthesis can limit bile acids influx. In *S*. Typhimurium and *E. coli*, bile acid exposure reduces OmpF synthesis while increasing OmpC synthesis. This shift in porin synthesis limits bile acid entry, because OmpF is a larger diffusion channel than OmpC.^150^ A similar mechanism is observed in *V. cholerae*, which expresses the narrower porin OmpU and represses the wider porin OmpT upon bile acid exposure.^151^ The rapid exchange of porins and other surface components that is needed when the pathogen transits from the stomach to the small intestine is facilitated by outer membrane vesiculation.^152^

Once bile acids have entered through the outer membrane or when encountering gram-positive bacteria, they reach the inner membrane and diffuse through it.^153^ Thus, active efflux mechanisms are needed to remove intracellular bile acids (Figure 5). The multidrug efflux pump AcrAB–TolC, expressed by numerous *Enterobacteriaceae*, including *Salmonella enterica* and *E. coli*, is the more extensively studied and is essential for resistance to various toxic molecules, including bile acids.^154^ Intracellular bile acids promote the expression of the transcriptional regulator *RamA*, which activates the transcription of *acrAB* and *tolC*.^155^ In *Campylobacter jejuni*, a homolog of AcrAB–TolC, CmeABC, confers resistance to various bile acids,^156^ while in *V. cholerae*, the efflux pump VexCD has an increased susceptibility to DCA and VexAB has a broad substrate specificity.^157^

Cytosolic bile acids can induce DNA damage. Therefore, DNA repair mechanisms, such as direct mismatch repair mediated by the DNA adenine methylase,^158^ nucleotide excision repair, and base excision repair ^159^ are essential for tolerating bile acids (Figure 5). By inflicting damage to the bacterial cell, the presence of bile acids activates stress responses. In *S. enterica*, the RpoS-dependent general stress response is required for bile resistance,^160^ while, in *E. coli*, the SOS response gene *dinF* protects against bile acids in reducing oxidative stress.^161^ In *Salmonella enterica* serovar Typhi (*S*. Typhi), exposure to bile acids leads to the production of reactive oxygen species. In return, the quorum-sensing system of *S*. Typhi increases the production of anti-oxidative enzymes.^162^

Finally, bacteria can shield themselves from bile by forming biofilms (Figure 5). Bile acids can induce or enhance the formation of biofilms in enteric pathogens, such as *V. cholerae*, opportunistic pathogens, such as *C. difficile*,^163,164^ or commensal bacteria, such as *Bacteroides fragilis* ^165^ or *Bifidobacterium*.^166^ By converting CA into DCA, the commensal *C. scindens* enhances biofilm formation by *C. difficile*, thus favoring its persistence and potentially increasing the risk of relapse.^164^ TCA disperses ingested mature *V. cholerae* biofilm in the gut, prior to activating the expression of virulence genes, thus favoring its colonization.^167^

#### Bile can act as an environmental signal for pathobionts and pathogens

Since bile acids and their metabolites are keystone features of the gut environment, many opportunists and pathogens use these cues to regulate expression of virulence factors that are needed for gut colonization.

Adherent invasive *E. coli* (AIEC) is a distinct pathotype of resident mucosa-associated bacteria that is enriched in CD patients compared to control subjects.^168^ AIEC take advantage of a specific intestinal environment to increase their replication and induce inflammation. In the lumen, bile acids promote the expression of AIEC virulence genes such as the flagellin *fliC* favoring bacterial persistence in the gut, as well as the long polar fimbriae *lpf* and the *gipA* factor facilitating bacterial interaction with and growth in Peyer’s patches (Figure 5).^122–124^ Moreover, the presence of bile salts activates secondary metabolic pathways allowing AIEC to use ethanolamine as a nitrogen source and propanediol as a carbon source, thus conferring a competitive advantage of these strains over other commensal bacteria.^125,126^ The utilization of propanediol by AIEC generates propionate that increases their virulence,^169^ but also synergizes with lipopolysaccharide to trigger IL-1β production and T_H_17 cell activation, thus promoting T cell-dependent intestinal inflammation.^127^ The OmpR transcriptional regulator, required for adhesion and invasion of AIEC in intestinal epithelial cells *in vitro*,^170^ is also involved in the resistance of AIEC to DCA.^171^

*S*. Typhimurium exposure to bile increases the activity of its type VI protein secretion system (T6SS) to deliver effector proteins with an antibacterial activity into adjacent cells, thus killing commensal bacteria and successfully colonizing the gut (Figure 5).^128^ Conversely, the expression of *Salmonella* pathogenicity island (SPI) 1 encoding the type III protein secretion system (T3SS) that injects effector proteins required for intestinal invasion and induction of ileitis, is repressed in presence of bile.^129^ The difference in regulation of these two secretion systems may favor bacterial infection by conferring a competitive advantage to *S*. Typhimurium against commensal enterobacteria in the bile acid-rich lumen, while delaying the expression of virulence genes required for intestinal invasion until the pathogen can reach the epithelium.^128,129^ In *S*. Typhi, the presence of bile upregulates the expression of genes encoded within SPI-1, thus increasing the invasion of the gallbladder epithelium.^172^ While the differences in regulation of SPI-1 between *S*. Typhimurium and *S*. Typhi are not fully resolved, they might be explained by the difference in stability of the dominant SPI-1 regulator, *hilD*, between these two strains in the presence of bile.^172,173^

In *V. cholerae*, primary bile acids increase virulence and motility,^174,175^ thus representing a signal for the bacteria to colonize the ileum. Finally, *C. difficile* induces a rapid influx of bile acids into the intestine during host colonization, which facilitates spore germination and outgrowth (Figure 5).^120^

#### The microbiota remembers past infections to better resist in the future

Infection with enteric pathogens disrupts the ileal absorption of bile acids and endocrine regulation of bile acids production (Figure 5).^23,24^ Oral or intravenous infection of mice with *S*. Typhimurium or *L. monocytogenes* respectively significantly reduces the expression of *Fgf15* in the ileum and its hepatic receptor components (*Fgfr4* and *Klb*).^23^ These changes are associated with hepatic pathophysiology and colonization of the hepatobiliary tract.^23^ In the porcine intestine, *S*. Typhimurium infection downregulates the expression of genes in the FXR pathway, reduces ASBT expression, upregulates the expression of NF-κB-dependent genes, and alters the expression of tight junction genes. These changes indicate a disruption of bile acid absorption.^24^ Thus, infection with invasive pathogens might alter FXR-FGF15 signaling and increase the flow of bile into the large intestine.

Interestingly, recent evidence suggests that the microbiota “remembers” prior infections to increase colonization resistance.^25^ Laboratory mice exhibit increased colonization resistance to *Klebsiella pneumoniae* several weeks after surviving an infection with the enteric pathogen *Yersinia pseudotuberculosis*.^25^ The underlying mechanism is that infection with *Y. pseudotuberculosis*, an enteric pathogen that invades ileal Peyer’s patches, increases the abundance of *Deltaproteobacteria* in the gut microbiota (Figure 5).^25^ *Deltaproteobacteria* are a class of bacteria metabolizing bile acid-derived taurine.^144^ Taurine consumption by *Deltaproteobacteria* results in the release of hydrogen sulfide,^176^ a gas that inhibits the growth of *K. pneumoniae* by aerobic respiration.^25,177^ Taurine supplementation mimics the effect of *Y. pseudotuberculosis* infection, suggesting that ileitis caused by the pathogen increases the flow of bile into the large intestine to promote growth of *Deltaproteobacteria*. An increased abundance of *Deltaproteobacteria* also enhances colonization resistance against *C. rodentium*,^25^ a pathogen that requires oxygen to grow in the gut environment.^178^ Supplementation with bismuth subsalicylate, a known sulfide sequestrant,^179^ impairs colonization resistance against *K. pneumoniae* in mice, ^25^ suggesting that microbiota-derived hydrogen sulfide limits growth of facultatively anaerobic opportunistic pathogens.

Although an increase in bile acid concentrations in the colon might be beneficial to enhance colonization resistance, excessive bile acid concentrations are associated with inflammatory disorders and colorectal cancer.^180^ Thus, a fine regulation of bile acid metabolism is required to enhance colonization resistance while limiting deleterious effects on the host.

## Bile acid malabsorption in CD

While in CD inflammation predominantly affects the terminal ileum and the colon but can occur anywhere in the gastrointestinal tract from the mouth to the anus, UC is characterized by a chronic inflammation restricted to the colon and the rectum.^181^

The terminal ileum constitutes the main site of active reabsorption of bile acids. Diarrhea due to malabsorption of bile acids and nutrients in the ileum is common in CD patients, while in UC patients, diarrhea results from a limited absorption of water and electrolytes through the damaged colonic mucosa.^182^ Vantrappen and coauthors were the first to demonstrate that CD patients, but not UC patients, present a reduced bile acid pool size compared to healthy subjects and that this decrease was inversely correlated with the Colitis Disease Activity Index.^183^ Similarly, Rutgeerts and coauthors revealed an increased turnover of primary bile acids in CD patients with ileal dysfunction and demonstrated that the severity of CA loss correlated with the extent of ileal disease, but also that affection of both ileum and colon lead to a total depletion of secondary bile acids.^184^ Over the years, several other studies confirmed that bile acid malabsorption occurs in CD patients with ileocolic disease, which is more severe after surgical resection of the distal ileum.^185–187^ The resulting increased flow of bile into the colon increases the bile acid pool, which can influence the gut microbiota composition.

Fecal samples of IBD patients exhibit dysbiosis compared to healthy subjects,^47^ which is associated with altered bacterial metabolism. Metagenomic analysis show that 12% of the microbial metabolic pathways changed between IBD patients and healthy subjects.^188^ Specifically, dysbiosis in IBD impairs microbial metabolism related to bile acids biotransformation, leading to an increase in primary bile acids and in sulfated bile acids and a decrease in secondary bile acids compared to healthy subjects.^49–51^ Interestingly, fecal dysbiosis and metabolite profiles also differ between CD and UC patients.^34,50,51^ Ileal CD patients exhibit a difference in microbiota composition, as well as a higher abundance of significantly altered microbial metabolic pathways and biological processes than CD patients without ileal involvement and UC patients.^188^ These variations may be explained by the different ability of the ileum to efficiently absorb bile acids in patients with ileal CD compared to IBD patients without ileal involvement, resulting in a different composition of the bile acid luminal pool reaching the colon, thus influencing the microbiota composition and its ability to produce various metabolites, among which bile acids.

In conclusion, bile acid malabsorption occurring in ileal CD may explain the differences observed by numerous studies in fecal dysbiosis, metabolomics, and inflammatory profiles between ileal CD patients and other IBD patients.

## Future directions and conclusion

Changes in the composition and function of the fecal microbiota have been linked to many non-communicable human diseases.^189^ Dynamic changes in the composition of gut-associated microbial communities reflect changes in microbial growth conditions.^190^ One of the key factors controlling microbial growth in the gut is the host environment.^191^ Bile is an important host-derived component of the gut environment. Bile acids and microbiota-derived bile metabolites influence the gut environment directly, by inhibiting the growth of some microbes, thereby contributing to colonization resistance against opportunistic pathogens. Furthermore, bile acids moderate the gut environment indirectly by maintaining mucosal epithelial barrier function and modulating innate and adaptive immune responses.

Consequently, changes in the composition or size of the bile acid pool disrupt gut homeostasis. For example, antibiotic-mediated depletion of microbes with 7-α-dehydroxylase activity changes the composition of the bile acid pool by decreasing the concentration of secondary bile acids, which favors an expansion of the opportunistic pathogen *C. difficile* in the fecal microbiota. A condition that changes the size of the bile acid pool is malabsorption in the ileum, which increases the flow of bile into the colon, and may explain the differences observed in fecal dysbiosis and metabolomic composition between ileal CD patients and UC patients. In turn, an increase in the bile acid pool in the colon provides *Deltaproteobacteria* with elevated amounts of bile-derived taurine to produce hydrogen sulfide, a metabolite that can exacerbate colitis.^143^

Thus, modulating the size and composition of the bile acid pool constitutes an interesting therapeutic approach to modulate intestinal inflammation and colonization resistance. For instance, fecal microbiota transplant is a key strategy in the treatment of recurrent *C. difficile* infection. However, the efficacy of microbiome reconstruction partly depends on the transplant's ability to restore BSH and 7-α-dehydroxylase functionalities and has been associated with an increased level of secondary bile acids and an increased signaling in the bile acid-FXR-FGF19 pathway.^192^ The bile acid-FXR-FGF pathway, as well as the bile acid-TGR5 pathway, play a major role in the maintenance of intestinal homeostasis. Agonists of these receptors may be of potential interest in the treatment of IBD. In mice, several FXR and TGR5 agonists showed an ability to improve symptoms of chemically induced colitis by reducing intestinal barrier permeability and inflammation.^73,74^ The fluoroquinolone ciprofloxacin, an antibiotic used in the treatment of IBD, is of potential interest because of its antibiotic and TGR5 agonist properties.^74^ Several FXR and TGR5 agonists are currently being evaluated in animal models and in clinical trials for treatment of hepatic and metabolic disorders.^193,194^ However, given the broad effect of bile acid receptors on the organism and contradicting reports on the role of secondary bile acids on inflammatory processes, further *in vivo* and clinical studies are needed to evaluate the potential benefits in the treatment of IBD. Finally, FXR-FGF15 signaling pathway is also modulated following bacterial infection of the gastrointestinal tract with bile-acid resistant pathogens.^23,24^ The recent discovery that bile acid-derived taurine can boost colonization resistance against opportunistic pathogens, such as *Klebsiella pneumoniae*, suggests that a bile metabolite-based approach could be an interesting strategy to enhance colonization resistance against various infections.^25^

In conclusion, the size and composition of the bile acid pool is an important modulator of gut associated microbial communities and could be targeted by approaches to remediate dysbiosis. Immunomodulation through precision edition of the microbiota, modulation of bile acid receptors using synthetic ligands, and microbiota-derived metabolites may become a powerful tool to restore intestinal homeostasis, improve FMT outcome, strengthen colonization resistance, and limit the recurrence of infection.
